# Construction and validation of a predictive model for the risk of peritoneal dialysis-associated peritonitis after peritoneal dialysis catheterization

**DOI:** 10.3389/fmed.2023.1193754

**Published:** 2023-09-15

**Authors:** Rong Dai, Chuyi Peng, Tian Sang, Meng Cheng, Yiping Wang, Lei Zhang

**Affiliations:** ^1^Department of Chinese Medicine, Anhui University of Chinese Medicine, Hefei, China; ^2^Graduate School, Anhui University of Chinese Medicine, Hefei, China; ^3^Department of Nephrology, The First Affiliated Hospital of Anhui University of Chinese Medicine, Hefei, China

**Keywords:** peritoneal dialysis, peritoneal dialysis-associated peritonitis, peritoneal dialysis catheterization, nomogram, predictive model

## Abstract

**Aim:**

To construct and validate a risk prediction model for the development of peritoneal dialysis-associated peritonitis (PDAP) in patients undergoing peritoneal dialysis (PD).

**Methods:**

This retrospective analysis included patients undergoing PD at the Department of Nephrology, the First Affiliated Hospital of Anhui University of Chinese Medicine, between January 2016 and January 2021. Baseline data were collected. The primary study endpoint was PDAP occurrence. Patients were divided into a training cohort (*n* = 264) and a validation cohort (*n* = 112) for model building and validation. Least Absolute Shrinkage and Selection Operator (LASSO) regression was applied to optimize the screening variables. Predictive models were developed using multifactorial logistic regression analysis with column line plots. Receiver operating characteristic (ROC) curves, calibration curves, and Hosmer-Lemeshow goodness-of-fit tests were used to verify and evaluate the discrimination and calibration of the prediction models. Decision curve analysis (DCA) was used to assess the clinical validity of the prediction models.

**Results:**

Five potential predictors of PDAP after PD catheterization were screened using LASSO regression analysis, including neutrophil-to-lymphocyte ratio (NLR), serum ALBumin (ALB), uric acid (UA), high sensitivity C-reactive protein (hsCRP), and diabetes mellitus (DM). Predictive models were developed by multi-factor logistic regression analysis and plotted in columns. The area under the ROC curve (AUC) values were 0.891 (95% confidence interval [CI]: 0.829–0.844) and 0.882 (95% CI: 0.722–0.957) for the training and validation cohorts, respectively. The Hosmer-Lemeshow test showed a good fit (*p* = 0.829 for the training cohort; *p* = 0.602 for the validation cohort). The DCA curves indicated that the threshold probabilities for the training and validation cohorts were 4–64% and 3–90%, respectively, predicting a good net gain for the clinical model.

**Conclusion:**

NLR, ALB, UA, hsCRP, and DM are independent predictors of PDAP after PD catheterization. The column line graph model constructed based on the abovementioned factors has good discriminatory and calibrating ability and helps to predict the risk of PDAP after PD catheterization.

## Introduction

Chronic kidney disease (CKD) has become a public health problem, affecting more than 10% of the global population (1). Peritoneal dialysis (PD) is an effective treatment for CKD (2). Peritoneal dialysis-associated peritonitis (PDAP) in patients with end-stage renal disease (ESRD) is one of the major complications of PD and can significantly increase the hospitalization and mortality rates of PD patients. Severe and prolonged PDAP may lead to peritoneal membrane failure and even death (3, 4). The incidence of PDAP in PD patients has been reported to vary widely by region, ranging from 11 to 73% (5).

Several risk factors of PDAP infection are present after PD catheterization in patients with CKD stage 5; however, the magnitude of the risk associated with each factor is unknown. A prediction model may be developed to determine the 1-year risk of PDAP before the insertion of the PD catheter. Such a model may be used to determine a measure of early treatment response that can improve the prognosis. Several investigators, including those from China and abroad, have explored the treatment outcomes for PDAP patients and have built visual prediction models (6–8). However, some factors in the aforementioned prediction models cannot be determined preoperatively, limiting their clinical application. Because of the possible associated adverse outcomes with PDAP, early detection of the PDAP risk is needed in clinical practice. Therefore, preoperative exploration of PDAP risk factors and targeted strategies to reduce the PDAP risk are essential for PD patients.

The aims of this study were to construct a clinical prediction model, visualize the model using column line plots, and finally validate it externally to provide individualized treatment plans for patients based on their 1-year PDAP risk.

## Materials and methods

### Study population

In this study, 376 patients admitted to Department of Nephrology, the First Affiliated Hospital of Anhui University of Chinese Medicine, with CKD stage 5 who underwent PD catheterization between January 2016 and January 2021 were selected. The study population was randomly split into a training cohort (*n* = 264) and a validation cohort (*n* = 112) at a ratio of 7:3 to establish and validate the prediction model. PDAP was diagnosed using the 2016 International Society of Peritoneal Dialysis (ISPD) guidelines for the treatment of peritonitis. The diagnosis of PDAP requires at least two of the following three items, confirmed by two physicians independently: abdominal pain or cloudy peritoneal fluid with or without fever; peritoneal fluid leukocyte count >0.1 × 10^9^/L and percentage of polymorphonuclear cells >50% (duration of abdominal stay ≥2 h); and positive microbiological culture of the permeate (9). The following patients were excluded from the study: (1) those with incomplete medical records, (2) those under the age of 18, (3) those whose dialysis effluent was not cultured, (4) those on immunosuppressant therapy, and (5) those with fungal or tuberculous peritonitis. The study was conducted in accordance with the principles contained in the Declaration of Helsinki and its subsequent amendments, and was approved by the ethics committee of the First Affiliated Hospital of Anhui University of Traditional Chinese Medicine (approval no.: 2021AH-73). All patients have signed the informed consent.

### Data collection

We recorded the clinical information (sex, age, education, and comorbidities) and laboratory parameters, including serum white blood cell (WBC) count, red blood cell (RBC) count, hemoglobin (Hb), platelet (PLA), neutrophil-lymphocyte ratio (NLR), RBC distribution width (RDW), alanine transaminase (ALT), aspartate transaminase (AST), serum preALBumin (PA), serum total protein (TP), serum ALBumin (ALB), blood urea nitrogen (BUN), serum creatinine (Scr), uric acid (UA), cystatin C (CysC), glucose, triglycerides (TG), total cholesterol (TC), high-sensitivity C-reactive protein (hsCRP), serum sodium, serum potassium, ALBumin-corrected serum calcium, serum phosphorus, blood magnesium, blood total carbon dioxide (TCO_2_), ALBumin-to-creatinine ratio (ACR), urinary total protein-to-creatinine ratio (TCR), and 24-h urine protein. All data were collected at admission. The study outcomes included the development of PDAP within 1 year of initiation of PD and was assigned a value of 0 (no PDAP) or 1 (PDAP).

### Sample size

The effective sample size in a prediction study is determined on the basis of the number of outcome events, i.e., at least 10 outcome events per variable (EPV) to ensure accuracy (10). To allow for five or fewer predictors in the final multivariate logistic regression model, a predicted training cohort of ≥250 patients was required. Our sample size and the number of outcome events exceeded those determined by the EPV method, suggesting that our predictions are reliable.

### Statistical analysis

In this study, missing data with a deletion rate greater than 20% were excluded. Imputation for missing data was considered if missing data were less than 20% (11, 12). Continuous variables conforming to normal distribution were expressed using $\bar{x}$±SD and compared between two groups using independent samples *t*-test. Non-normally distributed data were expressed using median with interquartile range (IQR) and were compared between groups using Wilcoxon test. Categorical data were expressed using composition ratio and compared between groups using *χ*^2^ test. Least Absolute Shrinkage and Selection Operator (LASSO) regression was used to screen for the risk factors. Variables included in the model were screened using the Akaike information criterion minimum principle (13). The optimal parameter (*λ*) in the LASSO model was selected based on the minimum criteria using 10-fold cross-validation with partial likelihood deviation on the *Y*-axis, log(λ) on the *X*-axis, Lambda.min and lambda1SE plotted as dashed vertical lines at the optimal values, and lambda1SE as the model optimal value. Based on the individualized prediction model of PD outcomes, a nomogram was constructed. According to the importance of the influence of the prediction variable on the outcome variable, different values were assigned to each of the pre-measured variables, and then the values of each prediction variable were summed, and then the probability of the outcome event was calculated by the function transformation between the summation value and the probability of the outcome event.

In this study, the prediction model was evaluated in terms of three main aspects: discrimination, calibration, and net clinical benefit. The area under curve (AUC) of the receiver operating characteristic (ROC) curve was used to assess discrimination. The discrimination of the prediction model refers to its ability to distinguish between patients receiving PD who did and did not experience PDAP. Calibration curves and Hosmer-Lemeshow goodness-of-fit tests were used to assess calibration. The calibration of the prediction model was defined as the agreement between the predicted and observed probabilities. Decision curve analysis (DCA) was used to assess the clinical utility. DCA is a method for evaluating the clinical usefulness of a prediction model by comparing the net benefit of making clinical decisions based on the model with the net benefit of making decisions based on other strategies. In our study, we performed DCA to evaluate the clinical applicability of the nomogram we developed for predicting the risk of developing PDAP in PD patients. Bootstrap method was used to repeatedly sample 1,000 times for internal validation of the model. Continuous variables were interpolated with mean for data with normal distribution, median for data with skewed distribution, and plural for categorical variables.

Statistical analysis was performed using SPSS (version 26.0; IBM Corp., Armonk, NY, USA) and R (version 4.1.3; R Foundation for Statistical Computing) software. Two-tailed *p* < 0.05 indicated that the differences were statistically significant.

## Results

### Comparison of basic characteristics of patients in the training and validation cohorts

**Figure 1:**
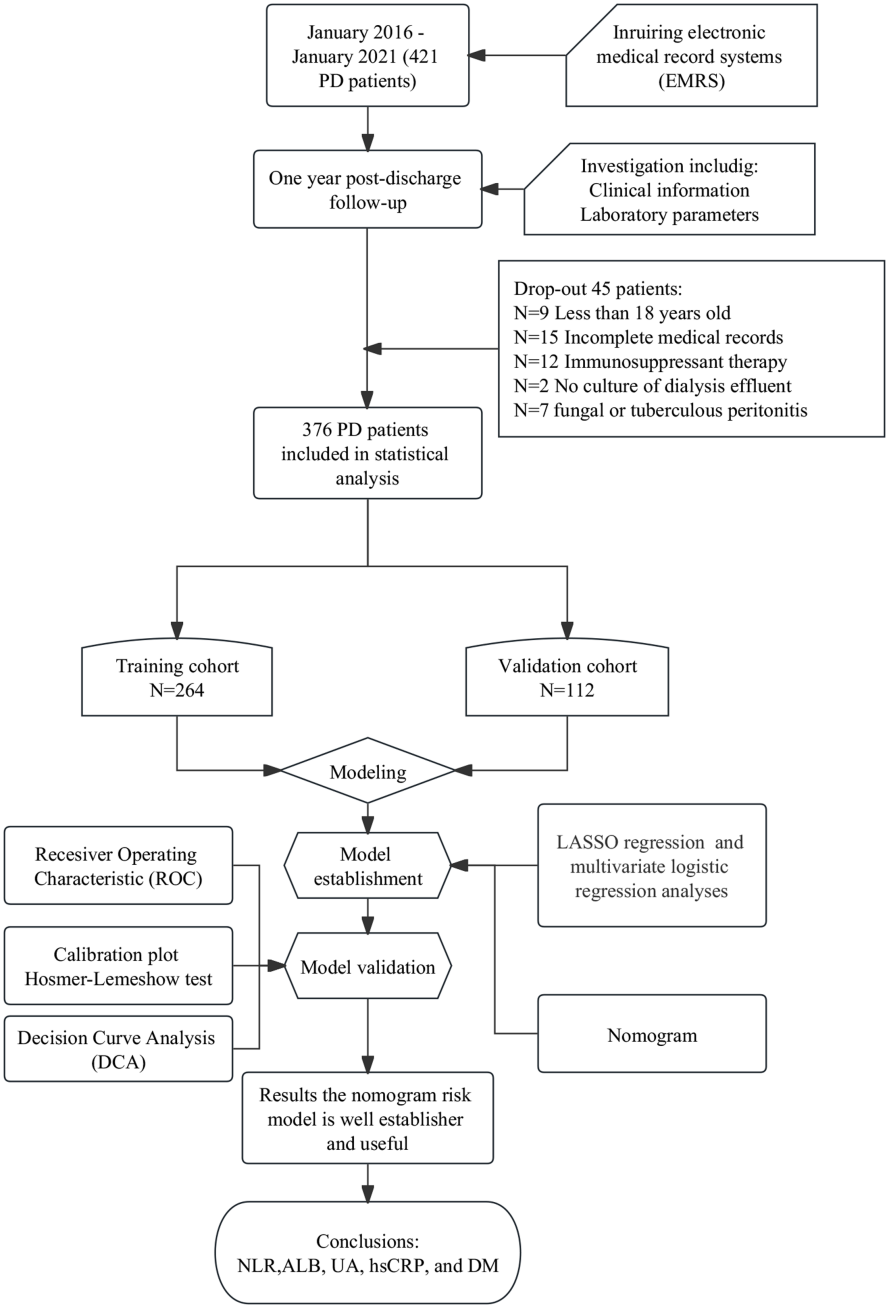
Flow chart of patient inclusion.

In total, 421 patients with CKD who underwent their first PD catheterization between January 2016 and January 2022 were identified. Of them, 376 PD patients with 1-year follow-up were screened and included in the final analysis: 264 (70.2%) patients were assigned to the training cohort and 112 (29.8%) to the validation cohort (Figure 1). There was no statistically significant difference in between the training and validation cohorts in terms of demographic characteristics, laboratory parameters, and comorbidities (all *p* > 0.05). Age, WBC, RBC, Hb, PLA, NLR, RDW, ALT, AST, PA, TP, BUN, Scr, UA, CysC, glucose, TG, TC, hsCRP, serum sodium, serum potassium, ALBumin-corrected serum calcium, serum phosphorus, serum magnesium, TCO_2_, ACR, TCR, and 24-h urine protein values were not distributed normally. Therefore, the median values of the aforementioned parameters were used as the cut-off to transform them into dichotomous variables. ALB level was grouped according to professional significance. The other independent variables were defined as categorical variables for analysis (Table 1).

**Table 1:** Clinical characteristics of the study population.

Index	Training dataset (264 episodes)	Validation dataset (112 episodes)	*p* value
Demographic characteristics			
Age (year)	54 (45,66)	51.5 (44.25, 64)	0.784
Education degree			
Primary school and below, n (%)	92 (34.8)	41 (36.6)	0.474
Above primary school, n (%)	172 (65.2)	71 (63.4)	0.354
Sex (male, *n*, %)	135 (51.1)	66 (58.9)	0.714
Laboratory examination			
WBC (10^9^/L)	6.11 (4.92, 7.49)	5.34 (4.22, 7.25)	0.206
RBC (10^12^/L)	2.72 (2.27, 3.21)	2.81 (2.53, 3.06)	0.650
Hemoglobin (g/L)	81 (67,95)	81 (72.25, 93.25)	0.742
Platelet (10^9^/L)	149.50 (113.25, 200.75)	133.00 (90.75, 201.25)	0.242
NLR	3.74 (2.37, 5.47)	3.47 (2.06. 5.26)	0.143
RDW-SD (fL)	44.65 (39.63, 48.97)	43.90 (38.95, 49.93)	0.449
ALT (U/L)	11 (8, 18.75)	13.5 (8.0, 20.25)	0.948
AST (U/L)	15 (13, 20)	15 (12.75, 21.00)	0.598
PreALBumin (mg/L)	291 (226.25, 329.00)	284 (234, 345.75)	0.904
Total protein (g/L)	59.45 (53.18, 65.07)	57.3 (50.28, 62.78)	0.552
Serum ALBumin (g/L)	34.6 (31.0, 37.8)	32.8 (30.13, 36.78)	0.674
Blood urea nitrogen (mmol/L)	32.63 (26.59, 40.47)	29.71 (21.98, 40.75)	0.871
Serum creatinine (μM)	821.55 (674.53,1000.30)	751.95 (511, 857.85)	0.408
Uric acid (μM)	477 (389.25, 564.75)	453.5 (379.5, 549.5)	0.121
Cystatin C (mg/L)	5.36 (4.65, 6.29)	5.32 (4.67, 6.74)	0.339
Glucose (mmol/L)	4.3 (4.00, 4.84)	4.57 (4.29, 5.00)	0.895
Triglyceride (mmol/L)	1.24 (0.89, 1.81)	1.17 (0.85, 1.00)	0.937
Total cholesterol (mmol/L)	3.97 (3.25, 4.61)	3.95 (3.55, 5.10)	0.509
hsCRP (mg/L)	2.38 (0.44, 10.33)	1.10 (0.43, 10.78)	0.265
Serum potassium (mmol/L)	4.59 (4.07, 5.18)	4.49 (3.95, 4.91)	0.527
Serum sodium (mmol/L)	141.1 (138.35, 142.95)	140.55 (137.65, 142.43)	0.402
Corrected calcium (mmol/L)	3.43 (3.01, 3.73)	3.47 (3.14, 3.75)	0.747
Serum phosphorus (mmol/L)	2.01 (1.58, 2.32)	1.93 (1.56, 2.42)	0.606
Serum magnesium (mmol/L)	1.00 (0.81, 1.17)	0.98 (0.89, 1.13)	0.904
Total carbon dioxide (mmol/L)	19.4 (16.6, 22.4)	20.8 (17.6, 22.6)	0.923
ACR (mg/g.CREA)	2331.17 (1206.44, 3741.20)	3997.76 (1886.26, 5524.49)	0.657
TCR (g/g.CREA)	3.38 (2.08, 4.99)	4.16 (2.30, 6.44)	0.453
24 h urine protein (g/24 h)	2.23 (1.25, 3.37)	2.87 (1.45, 4.43)	0.941
Combined disease			
Diabetes mellitus	99 (37.5)	42 (37.5)	0.342
Hypertension	233 (88.3)	105 (93.5)	0.986
Cerebral infarction	22 (8.3)	9 (8.0)	0.116

### LASSO regression for screening variables

**Figure 2:**
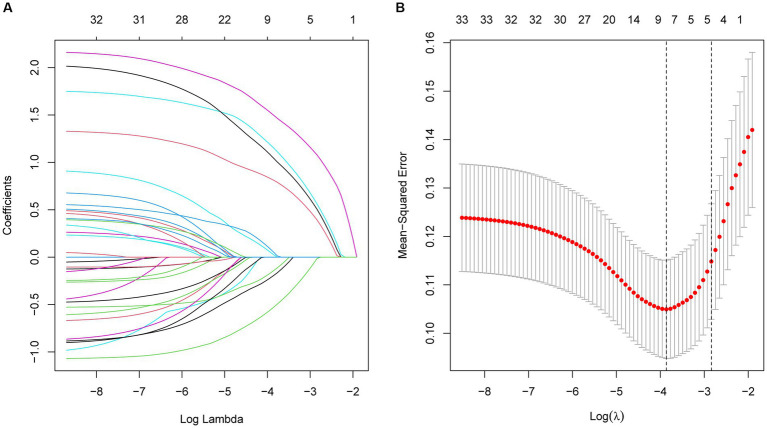
**(A)** Optimal parameter (λ) selection in the LASSO model, with the optimal tuning parameter logλ in the horizontal coordinate and the regression coefficient in the vertical coordinate. **(B)** Distribution of LASSO coefficients for the five factors, with the optimal tuning parameter logλ in the horizontal coordinate and the binomial deviance in the vertical coordinate.

Based on the clinical information, laboratory parameters, and prognosis of patients in the training cohort, dimensionality reduction was performed for the 34 variables by LASSO regression. Thus, the five most representative predictor variables were identified (Figure 2). The screened predictors included NLR, ALB, UA, hsCRP, and diabetes mellitus (DM).

### Construction of a clinical prediction model and column line plot

**Figure 3:**
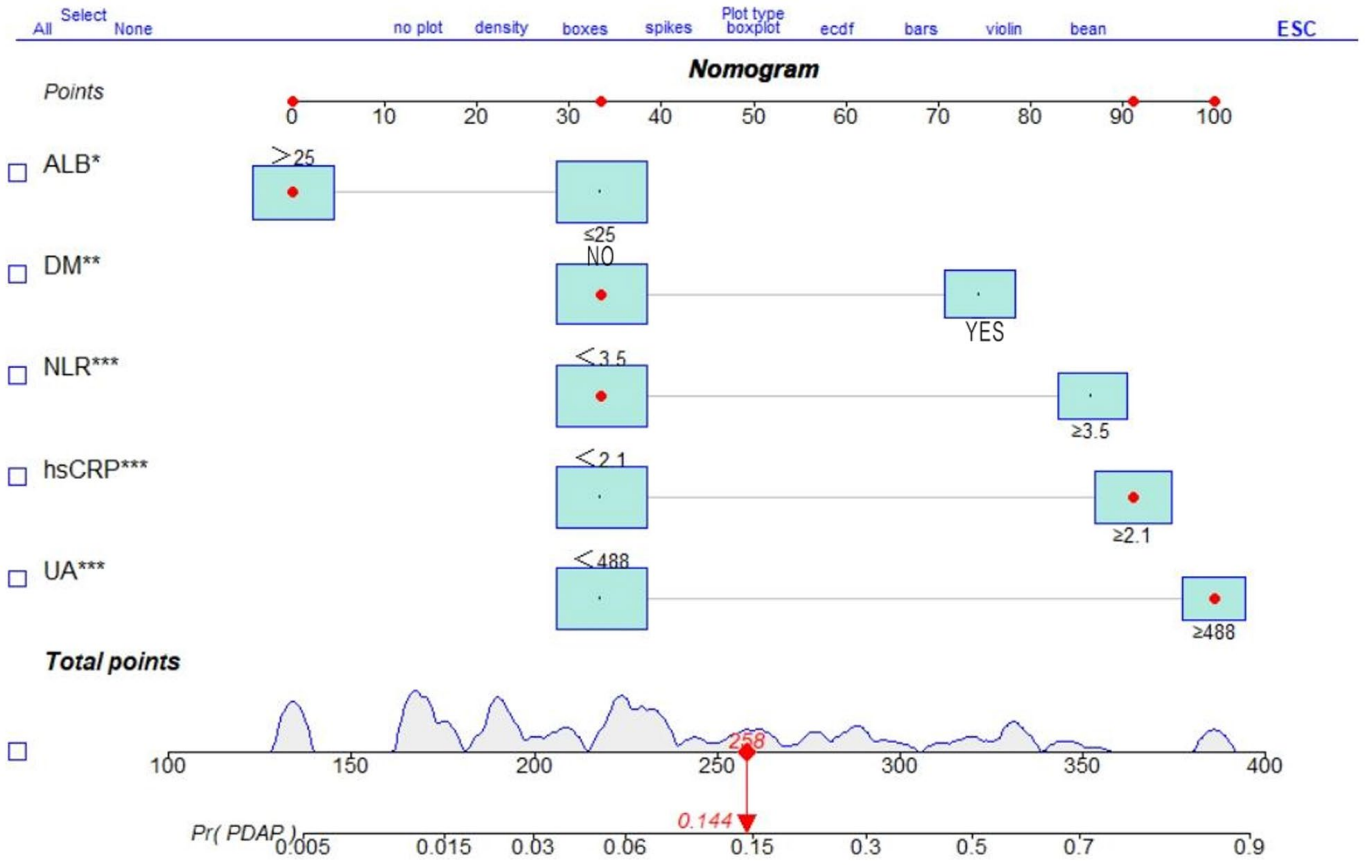
Nomogram of a clinical prediction model for the occurrence of PDAP after PD catheterization.

A multifactorial logistic regression prediction model was constructed using the development of PDAP in PD patients in the hospital and within 1 year after discharge as the dependent variable (not having PDAP = 0; having PDAP = 1). Five predictor variables were screened using LASSO regression analysis as independent variables (assigned values shown in Figure 2). The results showed that NLR, ALB, UA, hsCRP, and DM were risk factors of PDAP (Table 2). A column line graph, nomogram, was drawn using the predictor variables (Figure 3). The column line plot allows visualization of the corresponding numerical score for each variable. By adding the numerical scores and calculating the total score on all scales, a vertical straight line can be drawn to determine the probability of PDAP in a particular patient. Based on the predicted probabilities, the patient is at low risk of having PDAP. This calculated value can be used for treatment planning and patient counseling.

**Table 2:** Multifactorial logistic regression analysis of factors influencing the occurrence of PDAP after PD catheterization.

Variables	Variables assignment	*β*	*SE*	*OR*	95%CI	*p* value
NLR	<3.5 = 0 vs. ≥3.5 = 1	1.705	0.369	5.501	(2.671, 11.328)	<0.001
ALB	>25 g/L = 0 vs. <25 g/L = 1	−1.179	0.394	0.308	(0.142, 0.666)	0.003
UA	<488 mmol/L = 0 vs. ≥488 mmol/L = 1	1.743	0.368	5.716	(2.778, 11.761)	<0.001
hsCRP	<2.1 mg/L = 0 vs. ≥ 2.1 mg/L = 1	1.663	0.378	5.273	(2.513, 11.064)	<0.001
DM	Yes = 0 vs. No = 1	1.199	0.363	3.317	(1.630, 6.752)	0.001
Constants		−4.380	0.497			

### Internal validation of the nomogram

**Figure 4:**
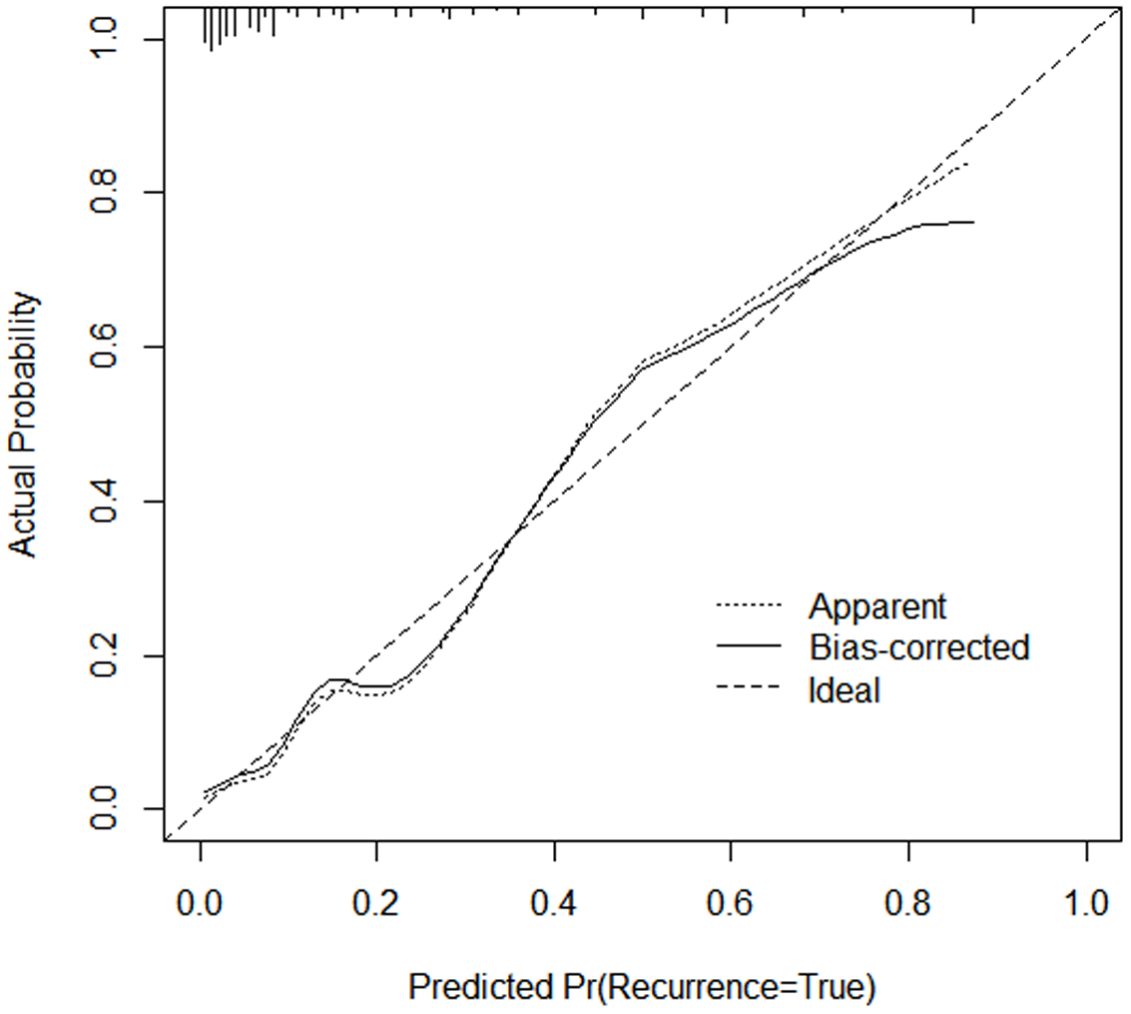
Internal calibration of the nomogram to predict the occurrence of PDAP after PD catheterization.

After 1,000 internal validation calibrations by Bootstrap method, its predicted risk profile of PDAP occurrence and the actual clinical risk profile of PDAP occurrence are still in good agreement (Figure 4).

### Validation and clinical utility of predictive models

**Figure 5:**
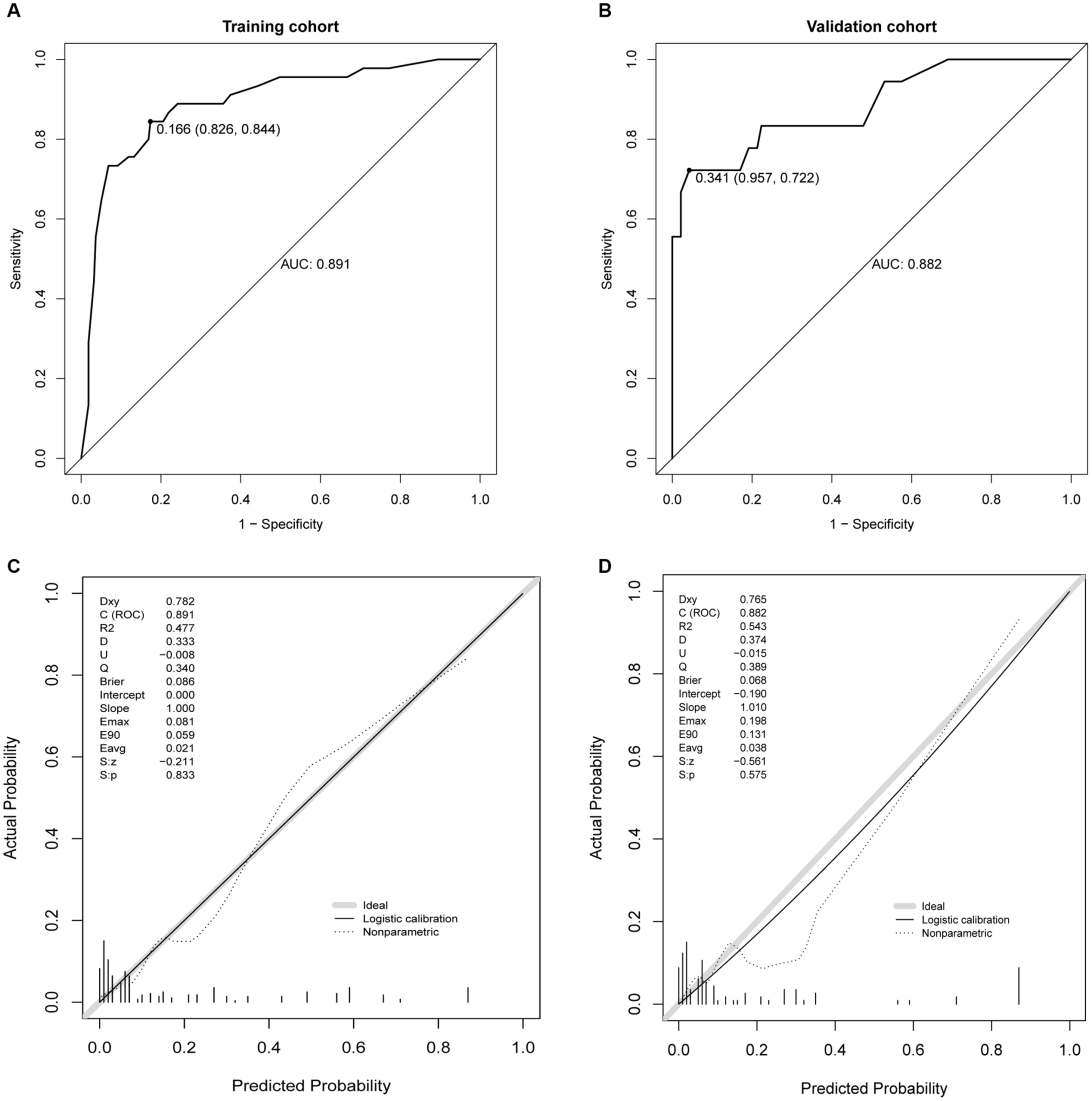
Receiver operating characteristic (ROC) curves. **(A)** ROC curve in the training cohort, **(B)** ROC curve in the validation cohort, **(C)** calibration plots in the training cohort, and **(D)** calibration plots in the validation.

**Figure 6:**
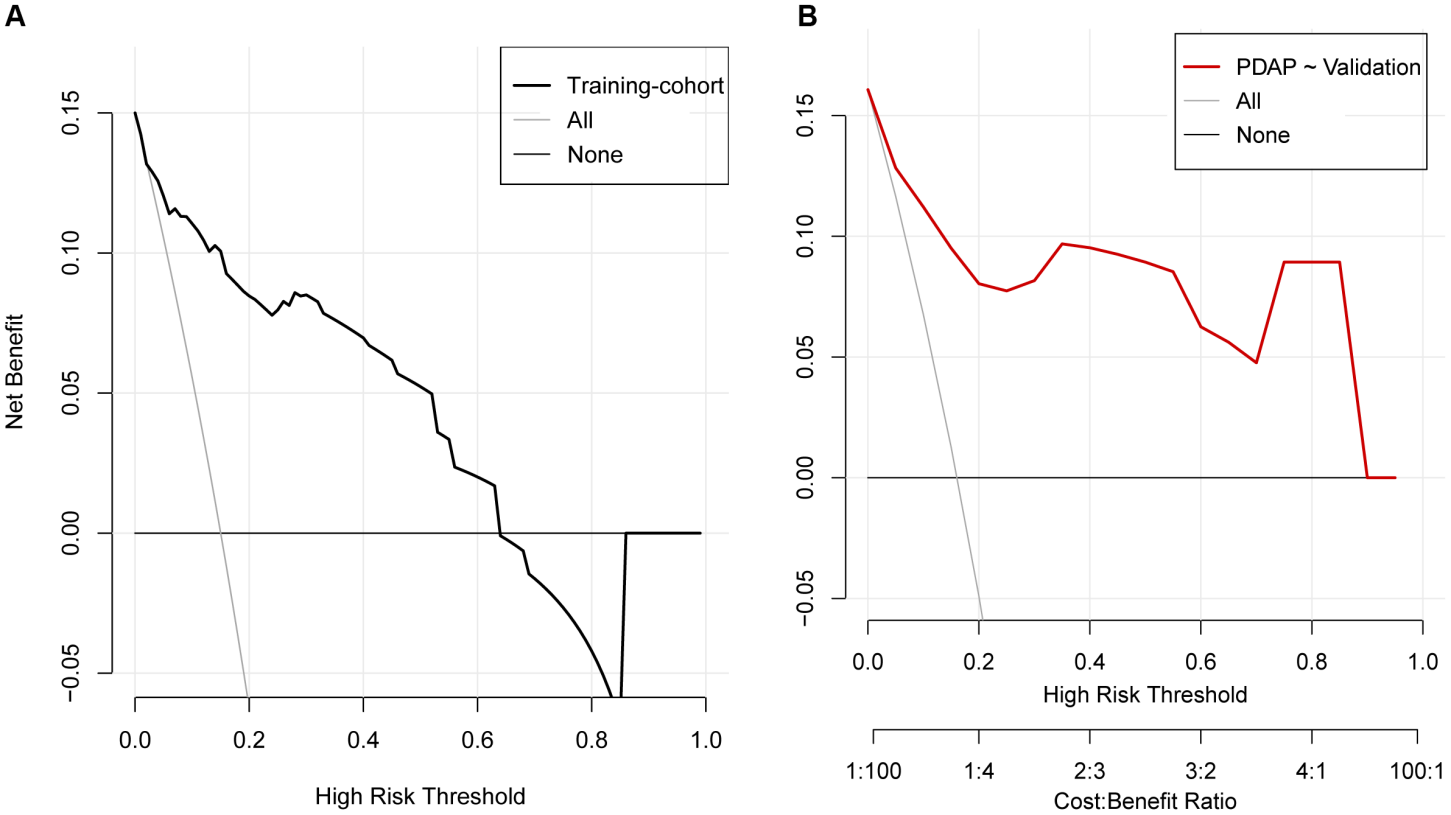
Decision curve analysis for the prediction model. **(A)** Training cohort, **(B)** validation cohort.

This study evaluated the prediction model in terms of discrimination, calibration, and net clinical benefit, which showed good accuracy for the prediction of the probability of having PDAP in PD patients with a C-index of 0.891 (95% confidence interval [CI]: 0. 0.829–0.844) for the training cohort and 0.882 (95% CI: 0.722–0.957) for the validation cohort (Figures 5A,B). Meanwhile, the Hosmer-Lemeshow test chi-square statistic showed that the calibration ability of the model was 5.06 (*p* = 0.829) for the training cohort and 7.34 (*p* = 0.602) for the validation cohort. The calibration curves showed a good fit for the training and validation cohorts, indicating that the predicted probability of PDAP within 1 year of PD initiation and the actual rate showed a good agreement (Figures 5C,D). The clinical decision curves for the training and validation cohorts showed a good net benefit (Figures 6A,B). The threshold probabilities, plotted on the x-axis, represent the range of appropriate risk probabilities (identified beforehand) at which a model could guide treatment when compared to the default strategies of “treatment for all” and “treatment for no-one.” When the 1-year PDAP risk was predicted using the column line plot, the net benefit of using the column line plot was significantly higher than those for “no intervention” and “full intervention” cohorts when the threshold probabilities for the training and validation cohorts were 4–64% and 3–90%, respectively, suggesting the clinical applicability of the lineage map. For our study, we aimed to determine appropriate thresholds that could be used to determine preventive treatment for PDAP. Treatment can refer to a variety of measures, including further examination or the initiation of targeted therapy. These data suggest that our line graphs are useful for clinical decision making.

## Discussion

Previous studies have mainly focused on disease etiology and treatment rather than on the development of prognostic methods, such as clinical prediction models. Many studies have explored whether a single variable (such as a biomarker or a new clinical biochemical parameter) can predict or correlate with certain outcomes, whereas building clinical prediction models by incorporating multiple variables is relatively complex. Nevertheless, clinical predictive models have been developed in recent years for various diseases (14, 15) including kidney disease (16). Clinical prediction models can inform patients and their physicians or other healthcare providers about the likelihood of disease development or progression in a patient, which can facilitate treatment decisions. The main risk factors included in previous PDAP clinical prediction models are age, UA, serum C-reactive protein (CRP)-to-Albumin ratio (CAR), NLR, RDW, DM, and cardiovascular disease (6, 17–21). The results of some previous studies are consistent with those of the current paper. However, most previous studies evaluated individual factors or postoperative indicators, whereas we identified preoperative indicators that are useful for clinical decision-making.

PDAP is the most serious complication of PD and leads to an increased risk of mortality and morbidity (22). Despite a significant decrease in the incidence of peritonitis, it still accounts for 41.3% of deaths and 20% of technical failures in PD patients (23, 24). This study retrospectively analyzed the clinical data of patients who developed PDAP over a 6-year period. For the first time, a prediction model based on the combination of NLR, ALB, UA, DM, and hsCRP was established to determine the 1-year PDAP risk of PD patients. The differentiation, accuracy, and clinical utility were validated in the validation set. Using the column line graph model, the individual risk of PDAP can be predicted preoperatively; this information may assist clinicians in making rational treatment decisions for PD patients.

A significant association was found between low serum ALB level at the time of initiation of continuous ambulatory peritoneal dialysis (CAPD) and the development of peritonitis (25). ALB is a major serum protein with important physiological functions, including maintenance of colloid osmotic pressure, binding to multiple compounds, and plasma antioxidant activity (26). Hypoalbuminemia is very common in patients with ESRD (27, 28). Notably, hypoalbuminemia is associated with increased morbidity and mortality in patients with PD (29). PD results in a significant loss of ALBumin, with protein leakage of up to 4.04 g/day during PD (30). In addition, low levels of serum ALB may result due to inflammatory response and malnutrition; it also increases the susceptibility of patients to infection (31). Ma et al. reported that hypoalbuminemia before the start of PD is a predictor of peritonitis (32). Therefore, hypoalbuminemia can be used as a warning sign for the development of peritonitis in patients undergoing CAPD and immediate intervention is needed to prevent peritonitis when serum albumin levels fall.

CRP is a marker of inflammation, and elevated serum levels are associated with an increased risk of cardiovascular events and mortality in the general population as well as in patients with kidney disease. Both CRP and ALB are useful prognostic markers for assessing mortality in patients with PD (33, 34). CRP levels reflect the severity of inflammation and ALB can be used as a nutritional marker in critically ill patients (35). CRP is an acute phase reactant mainly produced by the liver in acute and chronic inflammation. Previous studies have shown that CRP is an important risk factor for increased cardiovascular mortality in patients with PD (36). Chen et al. found that CRP was an independent predictor of increased all-cause mortality and risk of major adverse cardiovascular events in PD patients (37). Serum CAR, a composite indicator of inflammation and nutritional status, has recently been identified as an independent prognostic indicator for patients receiving treatment (35, 38). Some studies have found advantages of using CAR alone instead of CRP or ALB: the levels of inflammatory markers (e.g., CRP and ALB) vary depending on the inflammation severity. In addition, CAR can be used to assess both inflammation and nutrition. The use of this method is expected to improve the accuracy of prognostic prediction compared to CRP or ALB alone (21).

NLR is obtained by dividing the peripheral blood absolute neutrophil count with the peripheral blood absolute lymphocyte count. NLR has recently been reported to be associated with inflammation in ESRD, including HD and PD patients. The survival rate of patients with ESRD has been estimated using NLR (39–41). In PD, inflammation may result from several underlying causes, including uremic microenvironment, infection, reduced clearance of pro-inflammatory cytokines, volume overload, oxidative stress, and other dialysis-related factors (42, 43). NLR is a readily available parameter obtained from complete blood counts that is closely associated with inflammation and was originally considered as a prognostic indicator for tumors (44). Several recent studies of PD patients have shown that NLR is moderately associated with inflammatory marker levels (e.g., CRP, IL6, and TNF-α); higher NLR is associated with a higher mortality rate (40, 45, 46). In PDAP, increased NLR is independently associated with increased risks of treatment failure and catheter removal. NLR is a convenient and inexpensive parameter that may be indicate a poor outcome in patients with peritonitis (6).

UA is the end product of purine metabolism in humans and has antioxidant and pro-inflammatory properties. It is associated with the development of oxidative stress-related diseases, such as CKD and cardiovascular risk (47). Hyperuricemia is a risk factor for kidney disease, DM, and hypertension. High levels of UA are associated with a pro-oxidant and pro-inflammatory state. In patients receiving PD, a positive relationship was shown between UA levels and mortality (48, 49). High UA levels were associated with an increased risk of all-cause mortality in patients with PD compared to moderate UA levels (50). UA may induce oxidative stress by activating NADPH oxidase, stimulating the renin-angiotensin system, and interfering with mitochondrial function (51). Second, UA may regulate the inflammatory response through a variety of cytokines (52). Finally, high UA levels may reduce residual renal function in PD patients, leading to increased all-cause mortality (53, 54). Higher UA levels are associated with an increased risk of clinical manifestations of diabetic nephropathy in patients with DM. In addition, UA is a strong predictor of diabetic nephropathy progression (55).

The prevalence of DM is increasing in the general population and diabetic nephropathy is now the leading cause of ESRD worldwide (56, 57). Challenges to the overall health of ESRD patients with DM who are receiving renal replacement therapy (e.g., dialysis) (58). In ESRD patients with DM, adequate vascular access for hemodialysis is often problematic. As a result, many patients may select PD for renal replacement therapy. There are concerns that diabetic patients may develop PDAP because of their immunocompromised status (59). Inflammation plays a critical role in the development of diabetes, and higher CRP level is a risk factor for developing DM (60, 61). Although several studies have evaluated the risk factors of peritonitis in diabetic non-PD patients (62), they have also reported that DM is a risk factor for PDAP, and poor glycemic control is a risk factor for catheter tunnel and exit site infection in diabetic patients at the time of initiation of PD therapy (63–65). DM is associated with higher all-cause mortality in PDAP patients (66).

To the best of our knowledge, this is the first line graph model that allows risk assessment for the development of PDAP in PD patients. Our study has several advantages: first, our findings provide a valuable reference for physicians who manage patients with PD. Second, the column line graph prediction model is intuitive and practical because all included variables are obtained routinely.

Our study also has some limitations: first, the study had a retrospective design, which may have introduced selection bias. Non-random selection of participants and incomplete data may impact the representativeness of the study population, thus limiting the generalizability of findings to broader populations. Additionally, the lack of consideration for potential changes in predictor variables during treatment is noteworthy. Treatment interventions could influence variables, potentially introducing bias into our results. Second, we did not consider the effects of changes in variables during treatment on outcomes. Finally, we included 264 cases in the training cohort and 112 cases in the validation cohort; however, prospective studies with larger sample sizes are required.

In conclusion, we constructed an intuitive and practical column line diagram, which can predict the risk of PDAP in PD patients and provide a reference for the individualized treatment of PDAP patients.

## Data availability statement

The original contributions presented in the study are included in the article/supplementary materials, further inquiries can be directed to the corresponding authors.

## Ethics statement

The studies involving humans were approved by the ethics committee of the First Affiliated Hospital of Anhui University of Traditional Chinese Medicine (approval no.: 2021AH-73). The studies were conducted in accordance with the local legislation and institutional requirements. The participants provided their written informed consent to participate in this study.

## Author contributions

RD and LZ: research idea, study design, manuscript writing, and visualization. CP and TS: data collection. RD and CP: data analysis. RD and MC: statistical analysis. YW and LZ: supervision or mentorship. All authors have read and agreed to the published version of the manuscript.

## Funding

This study was funded by grants of the National Natural Science Foundation of China (82004165, 81673931, and 82274307), Natural Science Research Project of Anhui Higher Education Institution (KJ2021A0546), and Collaborative public relations project plan of Chinese and Western medicine for major and difficult diseases in Anhui Province (Approval number: 2021–70).

## Conflict of interest

The authors declare that the research was conducted in the absence of any commercial or financial relationships that could be construed as a potential conflict of interest.

## Publisher’s note

All claims expressed in this article are solely those of the authors and do not necessarily represent those of their affiliated organizations, or those of the publisher, the editors and the reviewers. Any product that may be evaluated in this article, or claim that may be made by its manufacturer, is not guaranteed or endorsed by the publisher.
